# Supplementary material for: FILM: Mapping organellar metabolism by mid-infrared photothermal modulated fluorescence
Source: ArXiv. 2026 May 4:arXiv:2504.04305v3. Preprint. [Version 3] (PMC13142552)
Supplement: Supplement 1 [file NIHPP2504.04305v3-supplement-1.pdf]

Table of Content

Supplementary Notes

Supplementary note 1: Pulsed mode enhanced signal by shifting high odd-order harmonics.

Supplementary note 2: Solvent background suppression with optical boxcar detection.

Supplementary note 3: Correlated noise in FILM images.

Supplementary note 4: Signal to noise calculation.

Supplementary note 5: Cosine similarity and Euclidean distance calculation.

Supplementary note 6: Lysosomes exhibit distinctive spectral features compared to the surrounding tissues.

Supplementary note 7: Investigation of the peak assignment of lysosomal signatures.

Supplementary Figures

Fig. S1: Comparison of fluorescence excitation light exposure durations.

Fig. S2: Time-domain and frequency-domain simulation of pulses with different duty cycles.

Fig. S3: Fluorescence thermal sensitivity measurement.

Fig. S4: Noise correlation in FILM images.

Fig. S5: FILM hyperspectral images with different IR frequency.

Fig. S6: Spatial feature verification of SPEND denoising process.

Fig. S7: FILM spectrum of eight standards for spectral deconvolution.

Fig. S8: Lysosomes exhibit distinctive spectral features compared to the surrounding region.

Fig. S9: Spectral phasor analysis of FILM hyperspectral data.

Fig. S10: Evaluation of the influence of dyes on the spectrum.

Fig. S11: Euclidean distance between data points calculated based on t-SNE and categorized into age groups.

Fig. S12: Data correction and calibration process for FILM spectra.

Supplementary Table

Table. S1: Information on standards for spectral deconvolution.

Table. S2: IR marker peaks of eight references.

Supplementary Videos

Supplementary Video 1: Hyperspectral FILM imaging of lysosomes before and after SPEND processing, along with the corresponding raw, uncalibrated spectra.

Supplementary Video 2: Fluorescence and hyperspectral FILM imaging, along with the corresponding raw, uncalibrated spectra of lysosomes in live *C. elegans*.

Supplementary notes:

Supplementary note 1: Pulsed mode enhanced signal by shifting high odd-order harmonics.

As depicted in **Extended Data Fig. 1**, mid-infrared photothermal signals generated by periodic IR pulses exhibit a low-duty-cycle characteristic, resulting in a spectrum rich in high harmonic content. This signal can be expressed as a Fourier series:

$$s(t) = \sum_{n=0}^{\infty} A_n e^{j2\pi nft}$$

Where, nf is the nth harmonic components. A_n is the Fourier coefficient, which reflects the amplitude of each harmonic. A lock-in amplifier (LIA) selectively detects only the first harmonic component.

Pulsed visible light can be described as a square wave, given by:

$$x(t) = \sum_{k=0}^{\infty} B_k \cos(2\pi k f_c t)$$

Where, kf_c is the kth harmonic components. B_k is the Fourier coefficient.

When the photothermal signal is gated by the pulsed visible light, the detected signal is given by:

$$d(t) = s(t) \cdot x(t) = \sum_{n=0}^{\infty} A_n e^{j2\pi nft} \cdot \sum_{k=0}^{\infty} B_k \cos(2\pi k f_c t)$$

$$= \sum_{n=0}^{\infty} \sum_{k=0}^{\infty} A_n B_k e^{j2\pi nft} \cos(2\pi k f_c t)$$

$$= \sum_{n=0}^{\infty} \sum_{k=0}^{\infty} A_n B_k \frac{1}{2} (e^{j2\pi(nf+kf_c)t} + e^{j2\pi(nf-kf_c)t})$$

Since the visible light is modulated at twice the frequency of the IR pulses (i.e., $f_c = 2f$), leading to

$$d(t) = \sum_{n=0}^{\infty} \sum_{k=0}^{\infty} A_n B_k \frac{1}{2} (e^{j2\pi(n+2k)ft} + e^{j2\pi(n-2k)ft})$$

From this expression, we observe that when $k = 1$, third harmonic component in $s(t)$ (i.e., $n = 3$) is shifted into the first harmonic band since $n - 2k = 1$. Similarly, fifth harmonic is shifted when $k = 2$ and higher harmonics follow the same pattern.

Principally, there is a trade-off: shorter pulses shift more higher harmonics, while the overall decrease in energy reduces the amplitude of each original frequency component

(**Supplementary Fig. 2**). As a result, within the "stable zone" the signal reaches its peak when the duty cycle is set to approximately 50% (**Fig. 2f**). If we maintain the average power while reducing the duty cycle, the ideal scenario would be to match the peak temperature. However, due to the low photon budget of silicon photomultiplier (SiPM), the detector easily saturates, disrupting demodulation and ultimately reducing the signal (**Extended Data Fig. 1b**).

Supplementary note 2: Solvent background suppression with optical boxcar detection.

We used HeLa cells labeled with LipiRed to mark lipid droplets as a model system. The cells were maintained in phosphate-buffered saline (PBS) solution, and the IR light was set to 1650 cm^{-1} and 1740 cm^{-1}, corresponding to the absorption peaks of bulky environmental contribution and lipid droplets (specific to the labeled objects), respectively. The fluorescence dynamics recorded under these conditions are shown in **Extended Data Fig. 2a**. Notably, there is a 1.1 μs delay between the thermal dynamics associated with the surrounding environment (1650 cm^{-1}) and those caused by the direct heating of lipid droplets (1740 cm^{-1}). Furthermore, the cooling rate of the bulky environment-induced dynamics is significantly slower than that of the lipid droplets. The distinct time delays and prolonged decay characteristics of these two components offer a way to differentiate the actual sample from the surrounding environment. The pulse pair serves as the gating windows to capture the time-resolved fluorescence signal, which is less sensitive to slow dynamic processes, leading to minimal differences between them (**Extended Data Fig. 2b**). This allows for the differentiation and suppression of the surrounding solvent environment's contribution.

Supplementary note 3: Correlated noise in FILM images.

FILM employs heterodyne detection, utilizing lock-in amplifier (LIA) to selectively detect and amplify signals at a specific modulation frequency while suppressing noise outside this bandwidth. However, the low-pass filter within the LIA introduces a delayed response, preventing the system from instantaneously tracking rapid variations in the input signal. Consequently, the measured signal at each pixel retains residual influence from its neighboring pixels, leading to temporal and spatial correlation in the detected image noise. Moreover, frequency mismatches between the heterodyne detection system and the reference frequency set in the LIA can further exacerbate this issue. When the detected frequency deviates slightly from the expected reference, the LIA may inadvertently mix signals from adjacent pixels, resulting in unintended signal leakage. This leakage manifests as a structured noise pattern in the image, where neighboring pixels exhibit correlated noise rather than remaining independent. As shown in **Supplementary Fig. 4**. There is an obvious tailing phenomenon between the

pixels in the fast-scanning direction of the image, indicating that they are not independent of each other.

Supplementary note 4: Signal to noise calculation.

To evaluate the SPEND denoising performance of FILM hyperspectral data (**Fig. 2c-d**), we compared the SNR from both the spatial (image-level) and spectral dimensions.

Image SNR was assessed using the hyperspectral image at 1711 cm^{-1}, calculated by the following formula.

$$SNR_{Image} = \frac{\mu_{signal}}{\sigma_{noise}}$$

Where, μ_{signal} represents the mean pixel intensity of lysosomes. σ_{noise} denotes the standard deviation of pixel intensities in a non-lysosome blank area, representing background noise.

Spectral SNR evaluates noise characteristics across the fingerprint for lysosomes. It was assessed in the spectral dimension with the following formula.

$$SNR_{Spectral} = \frac{P_{spectral}}{\sigma_{spectral}}$$

Where, $P_{spectral}$ is the mean intensity of lysosomes at 1711 cm^{-1}, which is the strongest peak frame. $\sigma_{spectral}$ represents the standard deviation of intensity values within the same area at 1764, 1777, 1782, 1788, and 1797 cm^{-1}, serving as a measure of spectral noise.

Supplementary note 5: Cosine similarity and Euclidean distance calculation.

To quantitatively assess the accuracy of spectral unmixing (**Fig. 2f-g**), we employed cosine similarity and Euclidean distance to compare the input spectra with reconstructed results.

Cosine similarity evaluates the angular similarity between two spectral vectors, measuring their directional alignment while ignoring magnitude differences. The cosine similarity between the original input spectrum S_{orig} and the reconstructed spectrum S_{recon} is calculated as:

$$Cosine\ similarity = \frac{S_{orig} \cdot S_{recon}}{\|S_{orig}\| \|S_{recon}\|}$$

Where, $S_{orig} \cdot S_{recon}$ is the dot product of the two spectra and $\|S_{orig}\|$ and $\|S_{recon}\|$ are their respective Euclidean norms. A more accurate LASSO unmixing results in a reconstructed spectrum closer to the original input spectrum, with a cosine similarity approaching 1.

Euclidean distance measures the absolute difference between the original input and reconstructed spectra at each wavenumber, given by:

$$d(S_{orig}, S_{recon}) = \sqrt{\sum_{i=1}^{n} (S_{orig,i} - S_{recon,i})^2}$$

Where, $S_{orig,i}$ and $S_{recon,i}$ are the spectral intensities at the ith wavenumber. n is the total number of spectral data points. A lower Euclidean distance indicates a closer match between the original and reconstructed spectra.

Supplementary note 6: Lysosomes exhibit distinctive spectral features compared to the surrounding tissues.

As shown in **Supplementary Fig. 8a**, we observed that at the IR wavenumber of 1587 cm^{-1}, the lysosome signal is stronger than at the 1649 cm^{-1}; whereas the surrounding background tissue, likely visualized by autofluorescence, exhibited the opposite trend, with higher intensity at 1649 cm^{-1}. **Supplementary Fig. 8b** shows the profiles represented by the orange lines in the two images of **Supplementary Fig. 8a**. The difference between these two profiles demonstrates two distinct IR absorption features in space. By segmenting the hyperspectral spectrum with spectral phasor (**Supplementary Fig. 9a-b**), lysosomes could be well identified from the surrounding tissues, and the resulting spectrum is shown in **Supplementary Fig. 9c**. The surrounding tissue exhibited three characteristic peaks at 1553, 1649, and 1741 cm^{-1}, corresponding to the amide II, amide I, and ester C=O vibrations of proteins and lipids, respectively[1]. In contrast, the lysosome spectrum concentrated around 1587 cm^{-1} and 1711 cm^{-1}.

Supplementary note 7: Investigation of the peak assignment of lysosomal signatures.

As shown in **Supplementary Fig. 8c**, we found that, compared to the Amide I peak of protein at ~1649 cm^{-1}, the main peak for AA locates at ~1587 cm^{-1}, which matches the lysosomal signature. This peak of AA reflects the antisymmetric deformation of NH_3^+ and the antisymmetric stretching vibration of COO^- bonds[2]. Lysosomes contain diverse hydrolases that digest macromolecules, breaking proteins into AA and lipid esters into free fatty acids (FFA). Upon opening peptide bonds in proteins, the exposure of additional NH_3 and COOH functional groups leads to an IR absorption shift from 1649 cm^{-1} to 1587 cm^{-1}. Similarly, the hydrolysis of lipid ester into FFA causes the stretching vibration mode of C=O red-shifting from 1741 cm^{-1} to 1711 cm^{-1}.

Supplementary Figures:

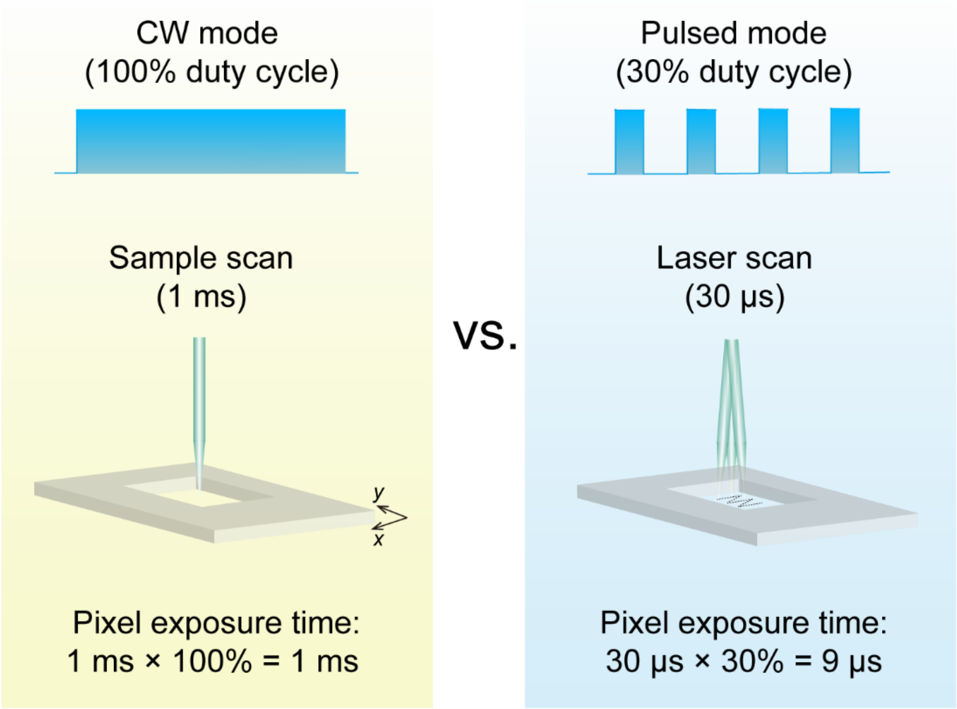

Fig. S1: Comparison of fluorescence excitation light exposure durations.

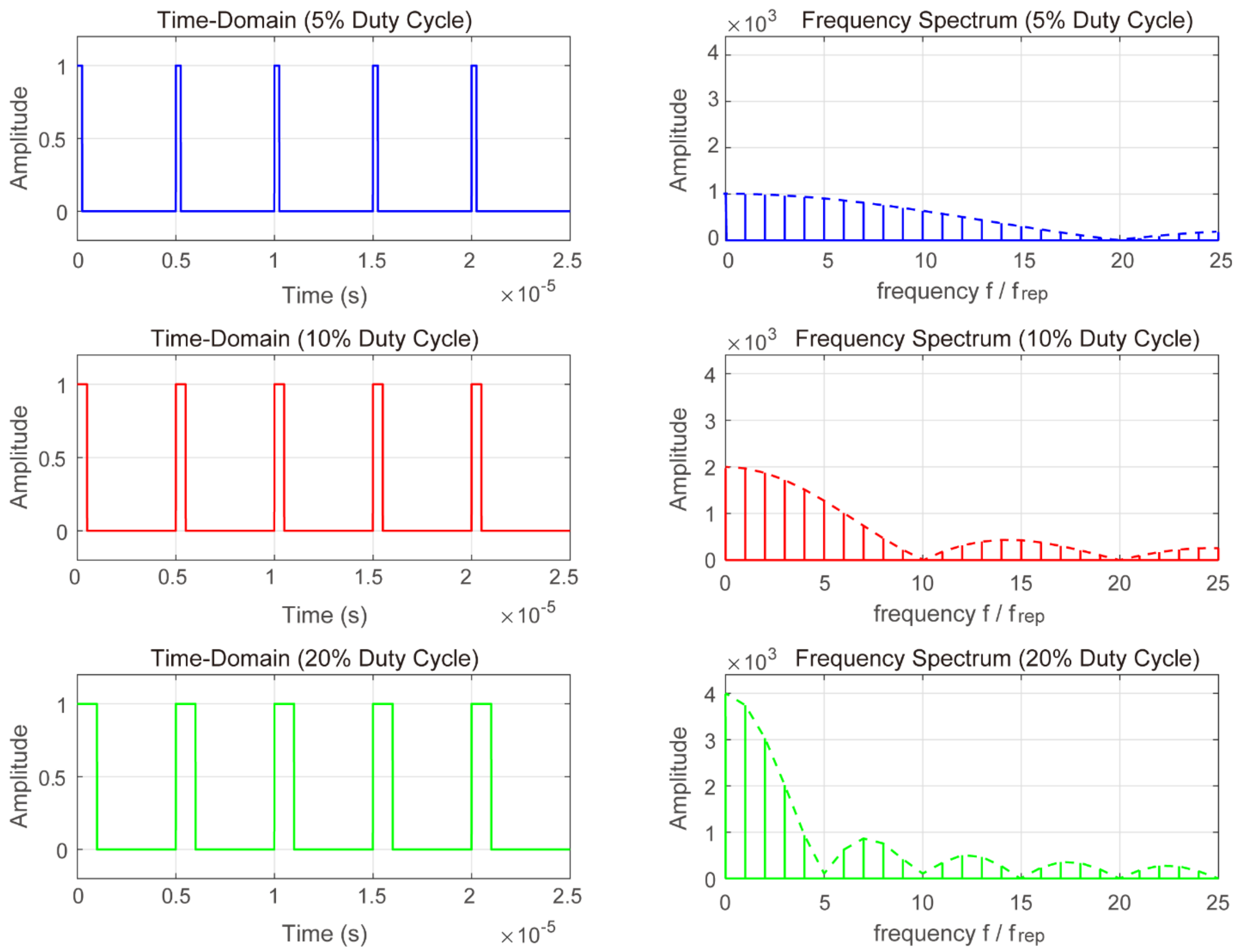

Fig. S2: Time-domain and frequency-domain simulation of pulses with different duty cycles.

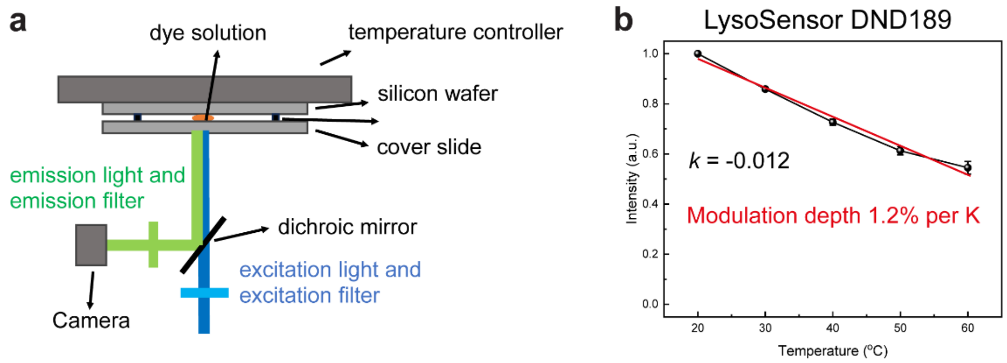

Fig. S3: Fluorescence thermal sensitivity measurement.

a, The system diagram of fluorescence thermal sensitivity measurement. **b,** Thermal sensitivity of LysoSensor DND189.

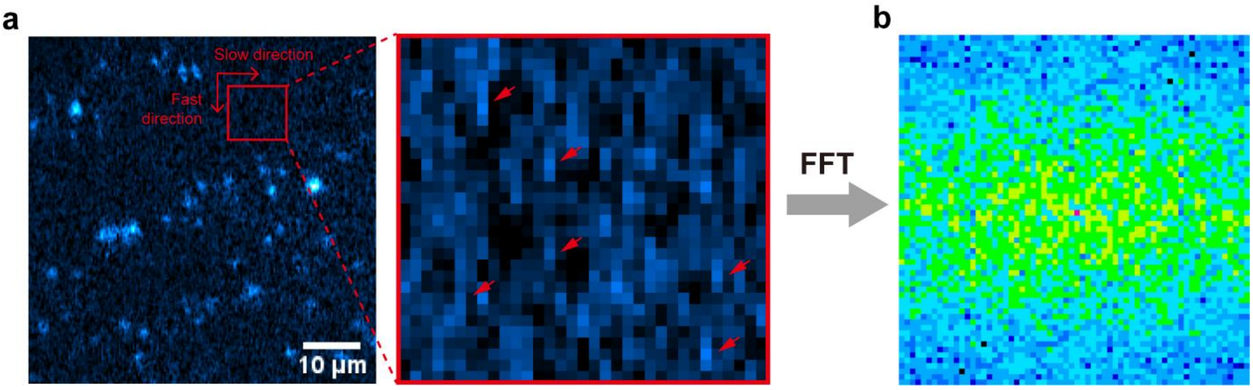

Fig. S4: Noise correlation in FILM images.

a, FILM image of lysosomes at 1711cm^{-1}. The red arrows indicate the correlation between pixels along the fast-scanning direction. **b,** Fourier spectrum of **a**. A clear decreasing trend from lower to higher frequencies along the fast axis suggests a structured noise pattern.

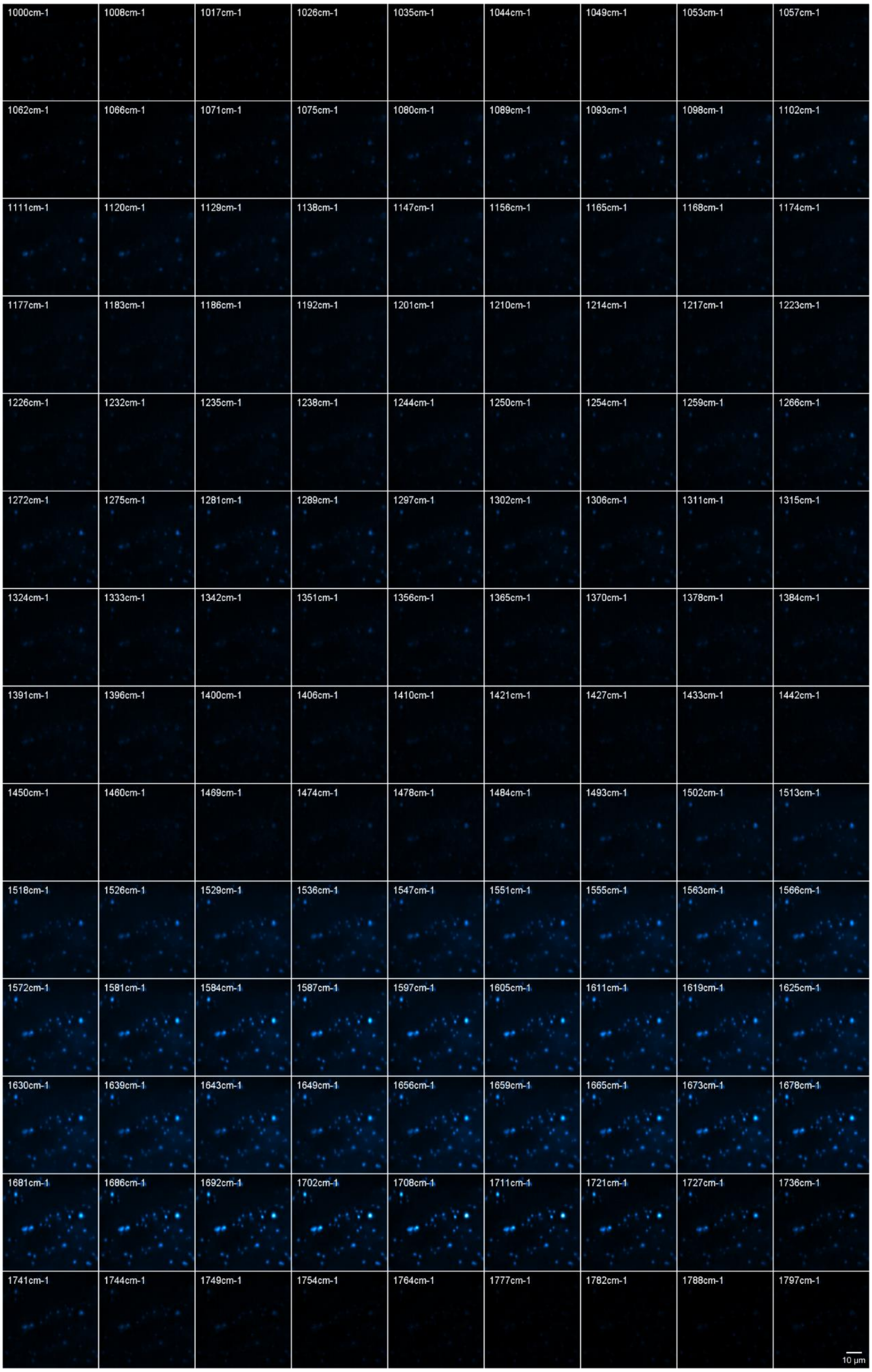

Fig. S5: FILM hyperspectral images with different IR frequency, highlighting the chemical selectivity.

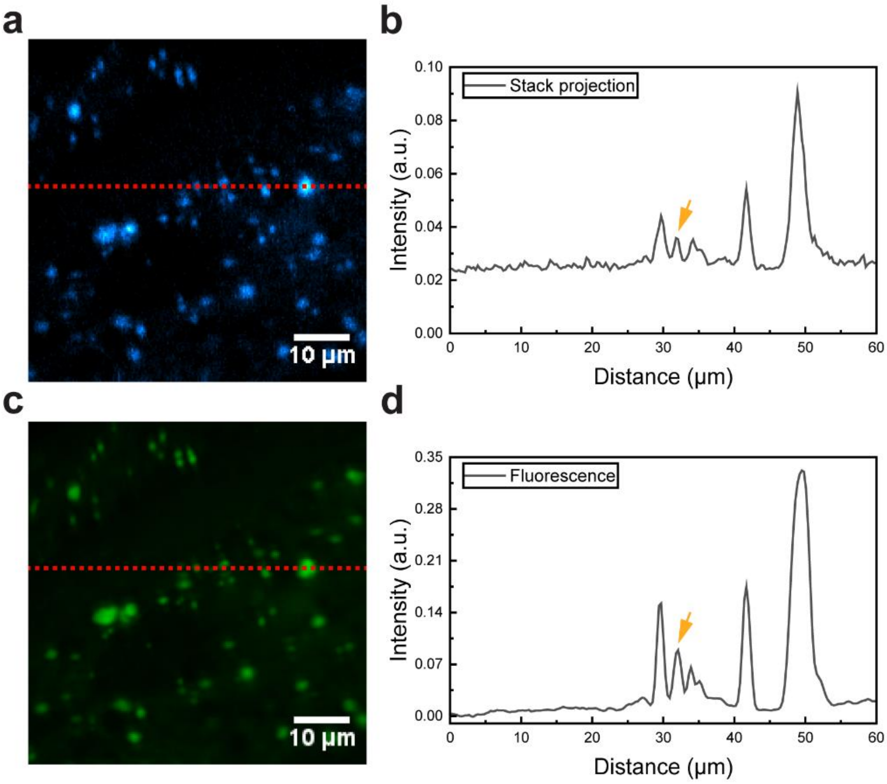

Fig. S6: Spatial feature verification of SPEND denoising process.

a, FILM hyperspectral imaging projection. **b,** Intensity profile across the red-dash line marked in **a**. **c,** DC fluorescence image. **d,** Intensity profile across the red-dash line marked in **c**.

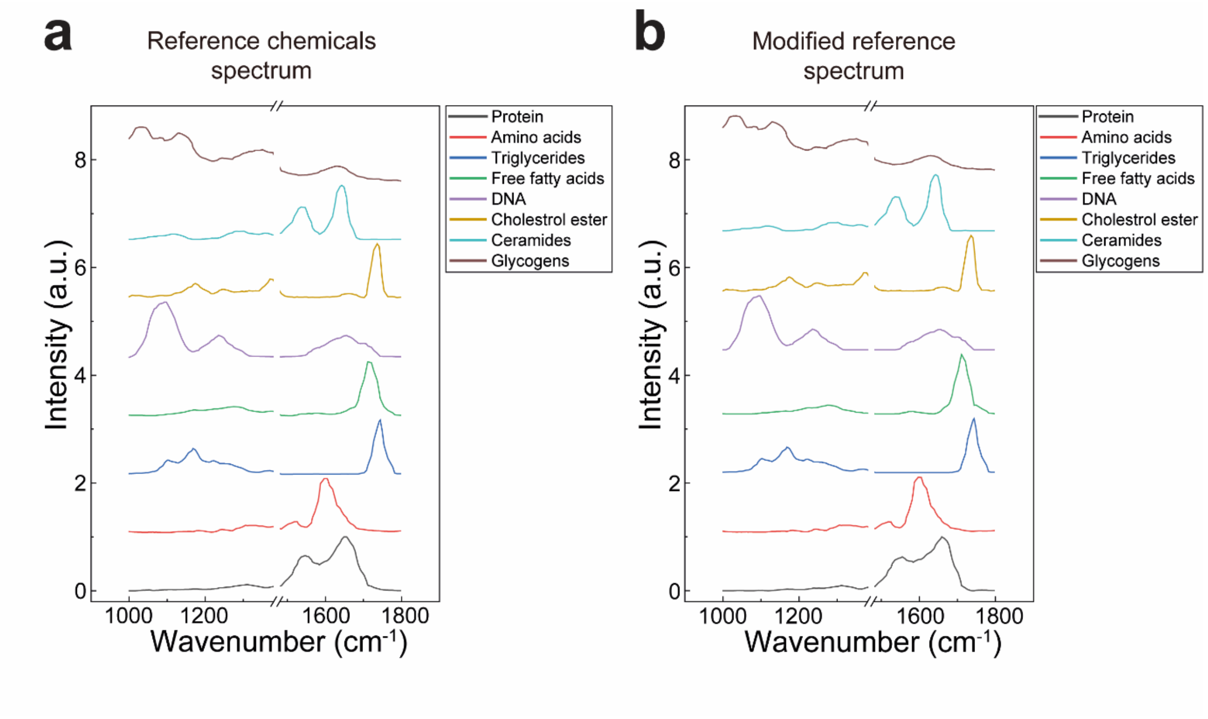

Fig. S7: FILM spectrum of eight standards for spectral deconvolution.

a, Reference spectrum of eight standards. **b**, MCR modified reference spectrum.

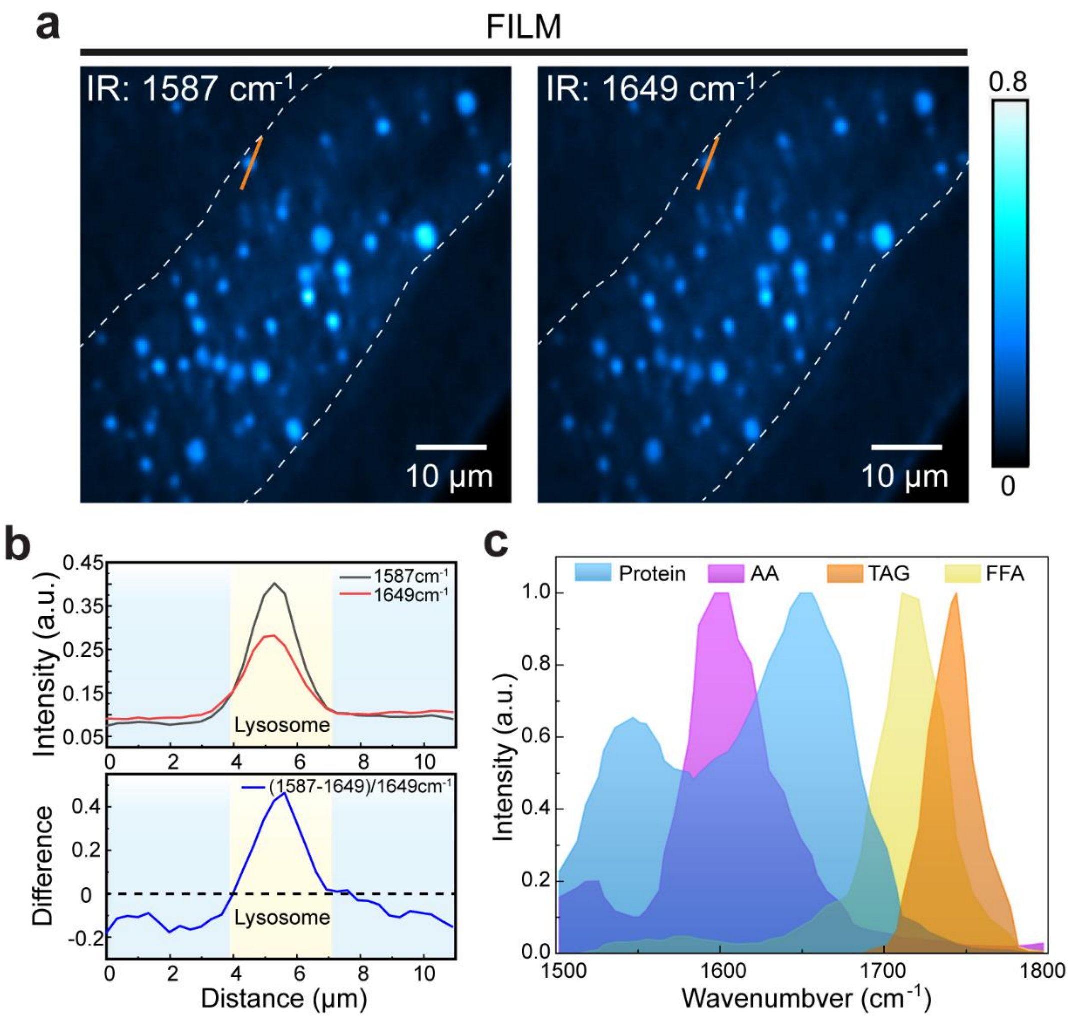

Fig. S8: Lysosomes exhibit distinctive spectral features compared to the surrounding region.

a, FILM images of *C. elegans* labelled with LysoSensor DND189. **b**, Intensity profiles along the orange lines marked in **a**. **c,** FILM spectrum of standard mixtures, including protein, amino acids (AA), triglycerides (TAG, lipid ester) and free fatty acids (FFA). These reference spectra were acquired with the same instrument and were used to assist in the assignment of characteristic peaks in lysosomal spectra. Scale bar: 10 μm.

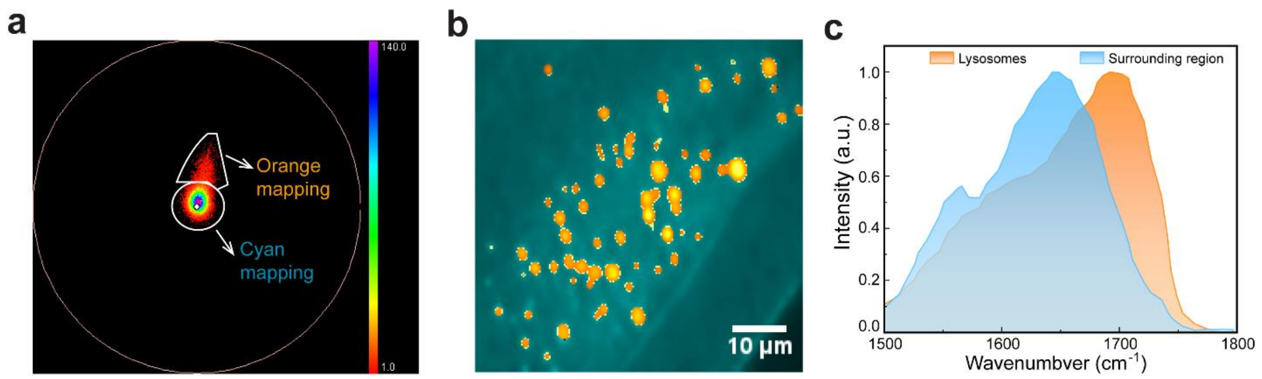

Fig. S9: Spectral phasor analysis of FILM hyperspectral data.

a, Spectral phasor plot on the hyperspectral image in **Supplementary Fig. 8**. **b,** Phasor segmentation map retrieved from the clusters marked in **a**. **c**, The spectral phasor analysis identified two distinct spectra in the FOV, corresponding to lysosomes and tissues, respectively.

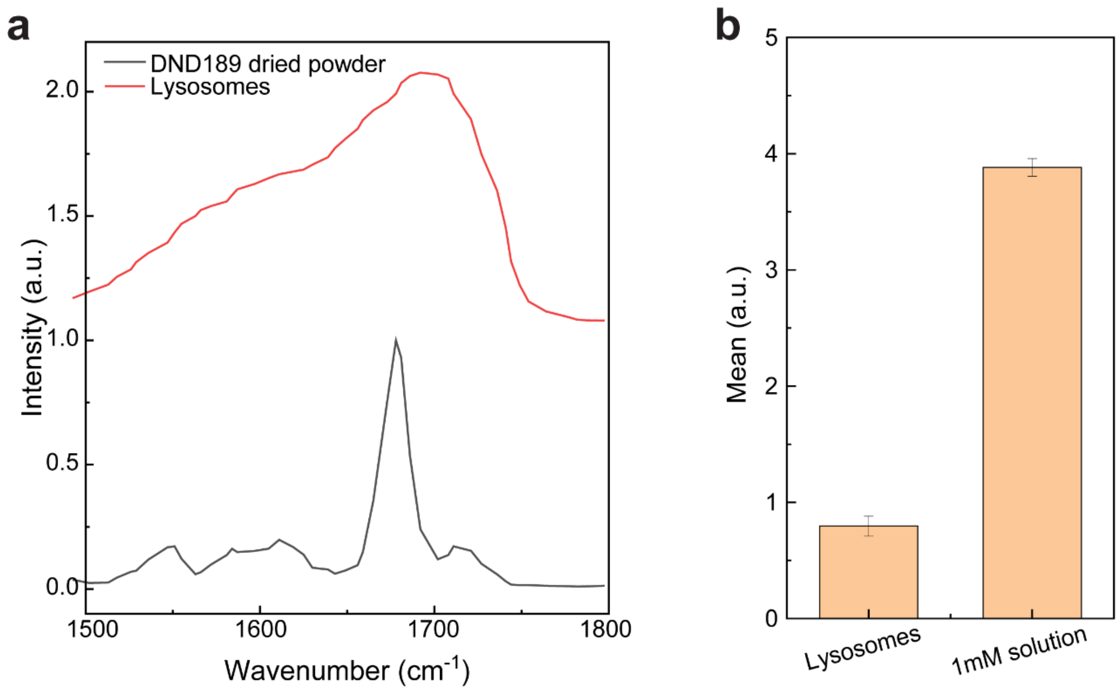

Fig. S10: Evaluation of the influence of dyes on the spectrum.

a, Comparison of lysosome spectra and LysoSensor DND189 dye. **b,** Fluorescence intensity comparison between lysosomes and standard 1 mM solution, indicating the dye concentration is below 1mM, which is lower than the concentration of the dominated biomolecules (n=8). Statistical data are presented as mean ± s.d.

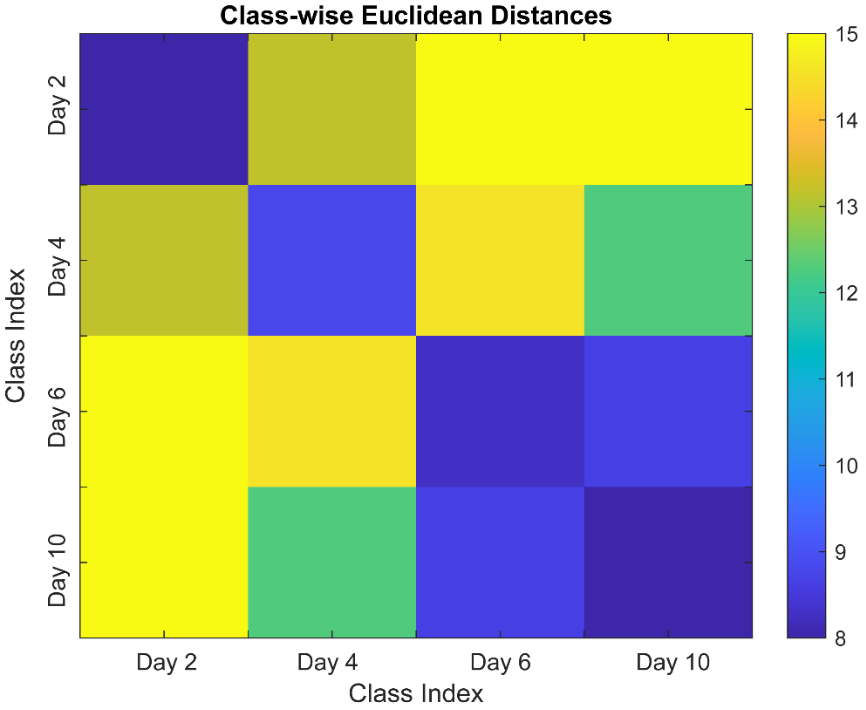

Fig. S11: Euclidean distance between data points calculated based on t-SNE and categorized into age groups.

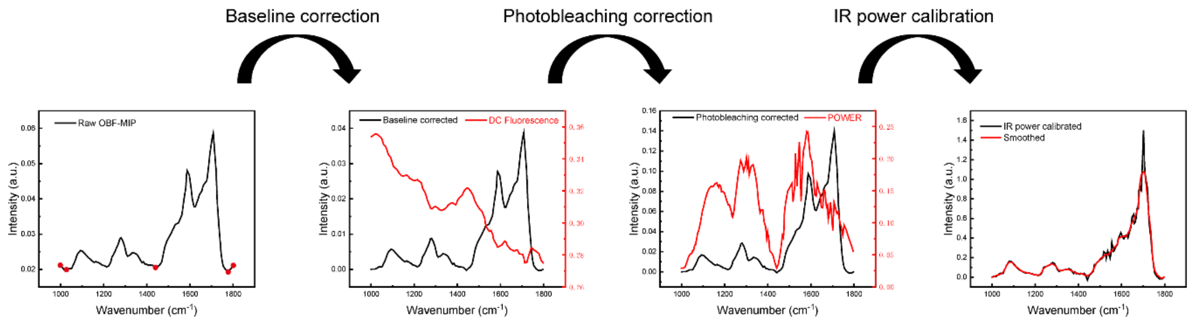

Fig. S12: Data correction and calibration process for FILM spectra, including baseline correction, photobleaching correction, and IR power calibration. The raw FILM spectrum (black) is baseline corrected, as indicated by the red dots, followed by photobleaching correction (red curve in the second panel), and then IR power calibration (red curve in the third panel) with smoothing applied. The red curve in the fourth panel represents the final output spectrum.

Table S1: Information on standards for spectral deconvolution

Chemicals	Composition	Suppliers
Triglyceride mixture	Triacetin (C2:0), Tributyrin (C4:0), Tricaproin (C6:0), Tricaprylin (C8:0), Tricaprin (C10:0)	17810, Supelco, Sigma-Aldrich
Protein mixture	Ribonuclease B, Insulin, Lysozyme, Transferrin, Bovine serum albumin (BSA), Trypsin inhibitor, β-lactoglobulin A, Carbonic anhydrase, Lactate dehydrogenase	MSRT2, Sigma-Aldrich
Ceramide mixture	L2-hydroxy C24:1, 2-hydroxy C18:1, C24:1, and C24:0 fatty acyl chains	22853, Cayman Chemicals
Fatty acid mixture	Lauric Acid (C12:0), Myristic Acid (C14:0), Palmitic Acid (C16:0), Stearic Acid (C18:0), Arachidic Acid (C20:0), Lignoceric Acid (C24:0), Palmitoleic Acid (C16:1), Oleic Acid (C18:1), Nervonic Acid (C24:1)	17942, Cayman Chemicals
DNA mixture	Sheared salmon sperm DNA solution, ~2000 bp average size	15632011, Sigma-Aldrich
Amino acid mixture	Alanine, Arginine, Aspartic Acid, Cystine, Glutamic Acid, Leucine, Lysine, Serine, Threonine, Tyrosine, Valine, Histidine, Isoleucine, Methionine, Phenylalanine, Proline, Glycine, and Ammonium Chloride	AAS18, Supelco, Sigma-Aldrich

Cholesterol ester mixture	Cholesteryl Palmitate (C16:0), Cholesteryl Oleate (C18:1)	C6072, Sigma-Aldrich<br>C9253, Sigma-Aldrich
Glycogen	Glycogen (from bovine liver), highly branched glucose polymer	G0885, Sigma-Aldrich

Table S2: IR marker peaks of eight references

Substance	Wavenumber (cm^{-1})	Assignments
Proteins	~1650	Amide I (C=O stretch; includes α-helix ~1650–1658, β-sheet 1618–1640/1680–1695, turn 1660–1680, random coil 1640–1650)
	~1550	Amide II (N–H bending + C–N stretching)
	~1309	Amide III (C–N stretch + N–H bend), α-helix–related region (~1310–1330)
Amino Acids	~1590	$\delta_{as}(NH_3^+)$ (asymmetric deformation); $\nu_{as}(COO^-)$ (asymmetric stretch)
	~1520	$\delta(NH_3^+)$ deformation (zwitterion) / aromatic ring modes
	~1310	ν(C–N) stretch with δ(NH) / CH bending (side-chain dependent)
Triglycerides	~1744	Ester C=O stretching (glycerol tri-ester)
	~1230	ν_{as}(C–O–C) asymmetric stretch of ester
	~1168	ν_s(C–O–C) symmetric stretch of ester
	~1098	ν(C–O) stretch of glycerol backbone / C–C skeletal vibrations
Free Fatty Acids	~1711	ν(C=O) of COOH
	~1280	ν(C–O) + δ(OH)_in-plane
	~1220	long-chain ν(C–C)/C–O combo & CH_2 wag/twist
DNA	~1655	Base vibrations (C=O in bases)
	~1225	$\nu_{as}(PO_2^-)$ asymmetric stretch (phosphate backbone)
	~1080	$\nu_s(PO_2^-)$ symmetric stretch (phosphate backbone)
Cholesterol Ester	~1735	Ester C=O stretching (cholesteryl ester linkage)
	~1375	CH_3 symmetric bending (acyl chains)
	~1175	ν_s(C–O–C) symmetric stretch of ester
Glycogen	~1340	δ(C–H) deformation
	~1130	C–O–C asymmetric stretching

	~1030	C–O stretching (C–OH + C–O–C symmetric) + ring breathing
Ceramides	~1640	Amide I (C=O stretch of amide linkage)
	~1540	Amide II (N–H bending + C–N stretching)
	~1285	C–N stretching + N–H bending / CH_2 wagging

Reference

1. Talari, A.C.S., Martinez, M.A.G., Movasaghi, Z., Rehman, S. & Rehman, I.U. Advances in Fourier transform infrared (FTIR) spectroscopy of biological tissues. *Appl Spectrosc Rev* **52**, 456-506 (2017).
2. Wolpert, M. & Hellwig, P. Infrared spectra and molar absorption coefficients of the 20 alpha amino acids in aqueous solutions in the spectral range from 1800 to 500 cm(-1). *Spectrochimica acta. Part A, Molecular and biomolecular spectroscopy* **64**, 987-1001 (2006).
